# Retinal Vessel Oxygen Saturation Measurement Protocols and Their Agreement

**DOI:** 10.1167/tvst.9.6.17

**Published:** 2020-05-19

**Authors:** Rebekka Heitmar, Robert P. Cubbidge

**Affiliations:** School of Life and Health Sciences, Aston University, Birmingham, UK

**Keywords:** retinal vessel oximetry, saturation, optical density, imaging, protocols, agreement

## Abstract

**Purpose:**

To assess agreement between different image sizes and analysis protocols for determination of retinal vessel oxygen saturation in the peripapillary retina of healthy individuals.

**Methods:**

Retinal oximetry measurements were acquired from 87 healthy volunteers using the IMEDOS Systems oxygen module. The peripapillary retinal vessels were assessed in a concentric annulus around the optic nerve head. Single and average vessel comparisons were made at different image field sizes of 30° and 50°. Comparisons between images obtained at 30° and 50° were made in a subset of 47 of the 87 individuals.

**Results:**

All subjects were normotensive and had normal intraocular pressures (9–16 mm Hg). Analyses of agreement between single vessel, averaged vessel, and between different size images were sought by Bland-Altman analyses, of which all yielded a low bias (<1% oxygen saturation). However, agreement between single vessels of consecutive images showed increased limits of agreement compared with saturation values calculated by averaging all or just the four major arcades of one image. Agreement between 30° and 50° images showed a similar bias as when comparing data obtained with the same camera angle setting but exhibited larger confidence intervals (arteries: bias = 0.21% [9.04/–8.62]%; veins: bias = 0.71% [14.82/–13.40]%).

**Conclusions:**

Averaging methods yielded the best agreement; there was little difference in average arterial and venous oxygen saturation between protocols, which analyze all vessels versus the four largest vessels. The least agreement was found for single vessel measurements and comparisons between different camera angles.

**Translational Relevance:**

Standardization of image capture protocols (same image size and undertaking a vessel averaging approach for oxygenation analysis) will enhance the detection of smaller physiological changes in eye disease.

## Introduction

Retinal hypoxia poses a threat not only to the retinal tissue but can also act as stimulus initiating neovascularization in ocular pathologies, such as diabetic retinopathy (DR), age-related macular degeneration, and vascular occlusions. Direct measurement of local retinal tissue oxygen saturation and metabolism is only possible using invasive methods,^1^ and is therefore not suitable for screening and follow-up monitoring in routine practice. Advancements in retinal imaging led to multiple custom built and commercially available devices, which allow indirect measurement of retinal vessel and tissue oxygen saturation parameters. The research pathway leading to noninvasive retinal oximetry measurements started in the mid-19th century with the discovery that hemoglobin exhibits a distinctly different absorption spectrum depending on the amount of oxygen bound.^2^ A more detailed review on the historic development of retinal oximetry can be found elsewhere.^3^ The two most widely used retinal vessel oximeters in the literature are commercially available devices employing a dual-wavelength photographic technology: the Oxymap T1 oximeter (Oxymap, Reykjavik, Iceland), and the oxygen module from IMEDOS Systems (Imedos Systems GmbH, Jena, Germany). The two devices differ with respect to wavelengths used and optical design. The T1 oximeter obtains two separate retinal images at 570 nm and 600 nm^4^ whereas the IMEDOS System uses a dual-wavelength filter to capture one single image.^5^

Retinal vessel oxygen saturation measurements using dual-wavelength retinal photography is a relatively new tool to assess the retinal metabolism in health and disease. Requiring only a dilated pupil to obtain a measurement, this technology has been implemented into clinical research examining patients with diabetes mellitus, cardiovascular disease, glaucoma, and high myopia.^6^^–^^8^ The findings of these studies have provided a valuable insight into the retinal oxygen metabolism showing how oxygen consumption is reduced in patients suffering from glaucoma^9^ and with increasing stages of DR.^10^ Other studies have shown a change in oxygen parameters depending on visual field loss in glaucoma^4^ and in hereditary conditions, including retinitis pigmentosa with characteristic loss of photoreceptors.^11^^,^^12^ As with any novel technique, which is aimed at clinical testing, it is paramount to have a robust protocol that has a low test-retest variability and high specificity and sensitivity.

Although there is still a lack of a standardized image analyses protocol, most researchers using the two instruments have employed a common approach. In brief, the main arterial and venous arcades are measured in a circular area around the optic nerve head (ONH),^5^^,^^13^ ideally on ONH centered images obtained with a camera angle of between 30° and 50°. Following multiple image capture (to ensure averaging across images is only done in images with comparable quality and illumination levels) either all or the four largest vessels passing through the measurement annulus are included in the calculation of arterial and venous oxygen saturation measurements. Some earlier publications have also used single vessel measurements in intervention studies and follow-ups,^14^ for example, in which quadrants around the ONH were compared.^13^ Although the literature examining device repeatability^5^^,^^13^^,^^15^ reports similar results, each device shows good agreement between measurements/images and standard deviations for both arteries and veins. A study comparing the two devices showed promising results with respect to agreement^16^ but highlighted the lack of concordance between devices meaning that measurements for long-term observations need to be collected by the same device. The lack of concordance was thought to arise because of the difference in wavelengths used to image the retina and also the algorithms employed to process the retinal images.

The aim of this study was to assess the agreement between (1) single vessel retinal oximetry measurements of two separate images (for arteries and veins separately); (2) average retinal oximetry measurements of two consecutive images (for arteries and veins separately); (3) average retinal oximetry measurements calculated by including all vessels versus just the four largest vessels (for arteries and veins separately); and (4) average retinal oximetry measurements calculated including the four largest vessels (for arteries and veins separately) obtained at different camera angle settings.

## Materials and Methods

The present study adhered to the Declaration of Helsinki after receiving a favorable opinion of the Aston University Ethics Committee. All subjects provided written informed consent following explanation of the nature of the study. We included 87 healthy nonsmokers (mean age range 19–52 years), but some of the analysis was only conducted on a subset of 47 individuals. The reason for this was because images with a 30° angle were captured for all 87 participants, whereas those with an image angle of 50° were only captured in 47 of the 87 participants. All participants were free from systemic disease, ocular abnormalities (including lenticular changes),^17^^,^^18^ and had no history of previous ocular surgery or trauma. All measurements were undertaken with the participants having abstained from caffeinated beverages, alcohol, chocolate, red meat, vitamin C, or participated in any form of exercise for a minimum of 4 hours prior to testing. Following intraocular pressure (IOP) measurements using noncontact tonometry (rebound tonometry using I-CARE, Midoptic, Birmingham, UK), one drop of tropicamide 1% (Minims; Chauvin Pharmaceuticals Ltd, Kingston upon Thames, UK) was instilled for pupil dilation. After resting in a sitting position for a minimum of 20 minutes to acclimatize to a room temperature of 22°C, baseline systemic blood pressure (BP) was measured using a digital BP monitor (UA-779; PMS Instruments, Maidenhead, UK) adhering to best practice guidelines. After these measurements, all participants underwent retinal vessel oximetry by dual wavelength photography as follows.

### Retinal Vessel Oxygen Saturation Measurements

Once full pupil dilation was reached, five images were obtained for each camera angle setting: that is, at 30° and 50° with the ONH centered. Oxygen saturation measurements were performed using the “oxygen tool” and VesselMap software Version 2 (IMEDOS Systems; IMEDOS GmbH) as described elsewhere.^5^ In brief, retinal images were taken with a customized dual wavelength filter (transmission bands at 548 and 610 nm; bandwidth 10 nm each) inserted in the illumination pathway of the instruments fundus camera (Zeiss FF450+; Carl Zeiss Meditech AG, Oberkochen, Germany). The retinal image is then recorded by a charge-coupled device digital camera, which captures a simultaneous image at the oxygen insensitive wavelength (548 nm) on the green channel of the camera, and the oxygen sensitive (610 nm) on the red camera channel.

**Figure 1.**
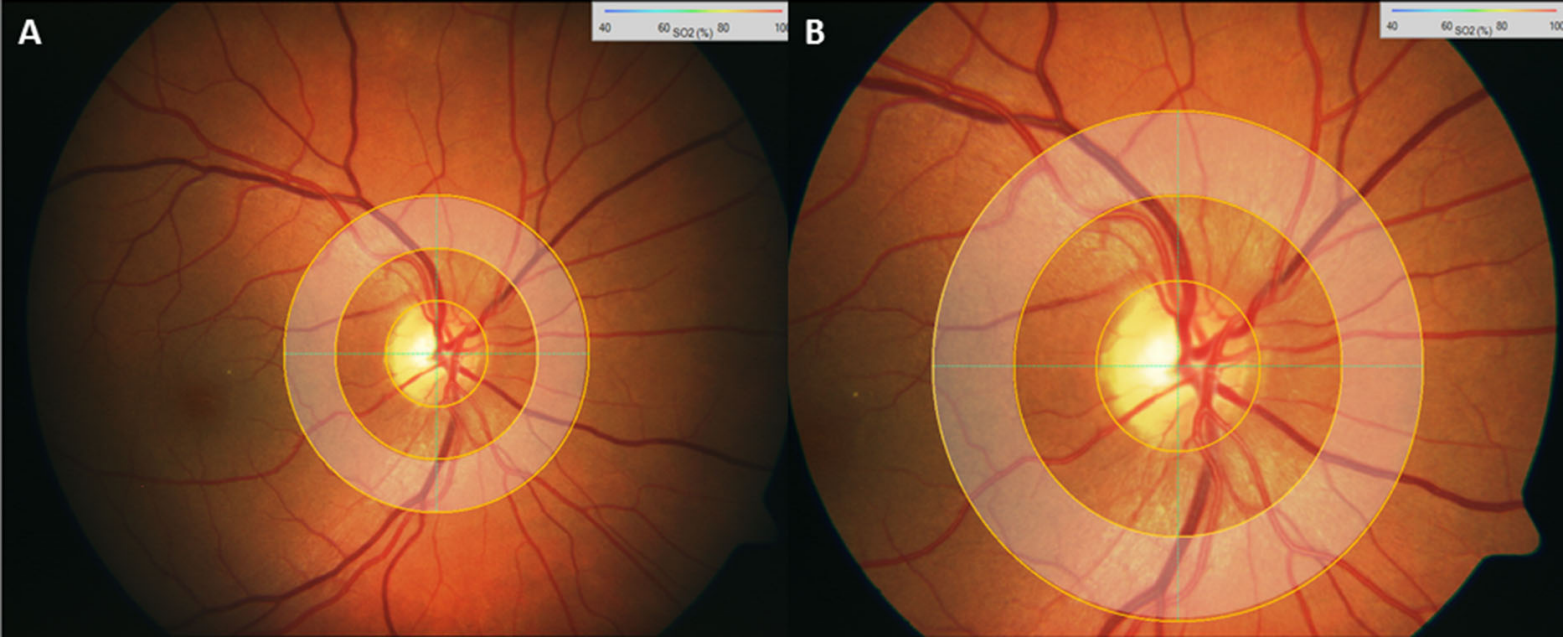
Illustration of the measurement area (shaded annulus) in the (A) 50° fundus image and the (B) 30° fundus image.

Optical densities of retinal vessels were measured as the logarithmic ratio of the fundus reflection at the vessel center and its surrounding tissue. The optical density ratio at 610 and 548 nm has been found to be inversely proportional to the vessel hemoglobin oxygen saturation when compensating for the vessel diameter and fundus pigmentation.^5^ The IMEDOS System allows image capture and analyses at 30° and 50° camera angle settings across the entire retinal image, within customized measurement zones or in a concentric measurement annulus around the optic disc. For the purpose of this study, the measurement area consisted of a concentric annulus around the ONH, which was half a disc diameter (DD) distant from the ONH and of one DD in width (Fig. 1).

### Data Analyses

Protocol a: Single vessel retinal oximetry measurements of two separate images.

For single vessel comparison we calculated the arterial and venous oxygen saturations for the four major vessels crossing the measurement annulus in each of the subset of 47 participants for which we have both 30° and 50° images (n_arteries_ = 188; n_veins_ = 188 for the single vessel analysis).

Protocol b: Average retinal oximetry measurements of two different images.

For this analysis, we calculated the average retinal arterial and venous oxygen saturation by including only the four largest vessels of each vessel category (i.e., the four largest arteries for arterial saturation, and the four largest veins for venous saturation) for two separate oximetry images.

Protocol c: Average retinal oximetry measurements calculated by including all vessels versus just the four largest vessels.

For this analysis, we calculated the average arterial and venous saturation by using the multimeasurement tool of the IMEDOS oxygen module, which allows for simultaneous measurement across multiple images at the exact same retinal location. To do so we selected three of the five images obtained and measured all arteries and all veins larger than 70 µ passing through the measurement annulus, and then calculated arterial and venous oxygen saturations first for all vessels selected, and second for the largest four vessels of each category.

Protocol d: Average retinal oximetry measurements calculated, including the four largest arteries and veins, obtained at different camera angle settings.

To compare images obtained at the 30° and 50° camera angles, we used the retinal arterial and venous oxygen saturations as calculated in (c), such that saturations were calculated by averaging values obtained of the four largest vessels of each category passing through the measurement annulus across three images (Fig. 1).

### Statistical Analyses

**Figure 2.**
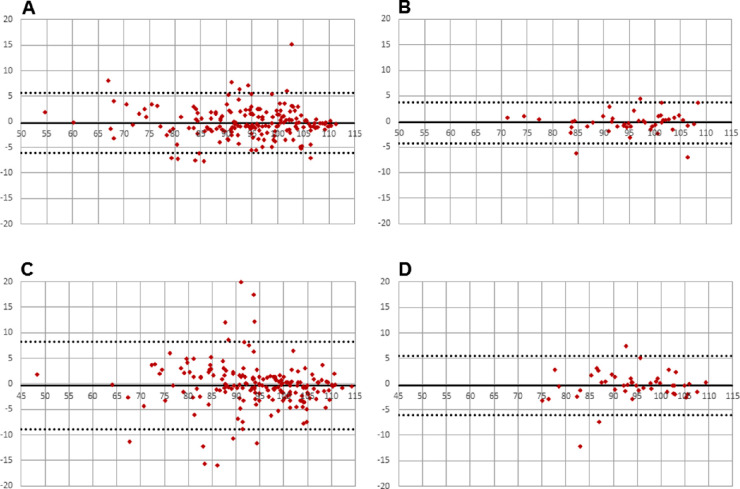
Intersession agreement (arteries). (A) Image 1 versus image 2 single vessels (30° camera angle); (B) average of four major vessels image 1 versus average of four major vessels image 2 (30° camera angle); (C) image 1 versus image 2 single vessels (50° camera angle); (D) average of four major vessels image 1 versus average of four major vessels image 2 (50° camera angle).

All data were normally distributed. Analysis of agreement was sought by Bland-Altman^19^ analysis using Microsoft Excel (Microsoft Corporation, Redmond, WA). Calculated outputs, that is, mean bias and limits of agreement, are presented in Figures 2^3^,^4^–5 and Table 1. Comparisons between single vessels, single images, and protocols were sought from a paired *t*-test (Table 2).

**Figure 3.**
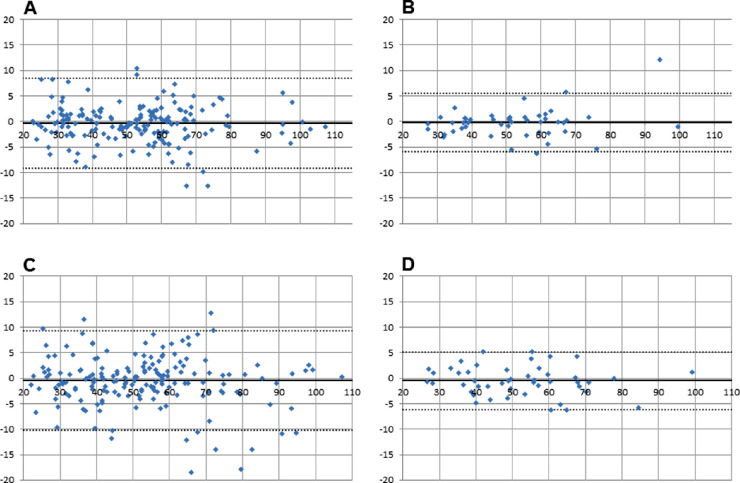
Intersession agreement (veins). (A) Image 1 versus image 2 single vessels (30° camera angle); (B) average of four major vessels image 1 versus average of four major vessels image 2 (30° camera angle); (C) image 1 versus image 2 single vessels (50° camera angle); (D) average of four major vessels image 1 versus average of four major vessels image 2 (50° camera angle).

**Figure 4.**
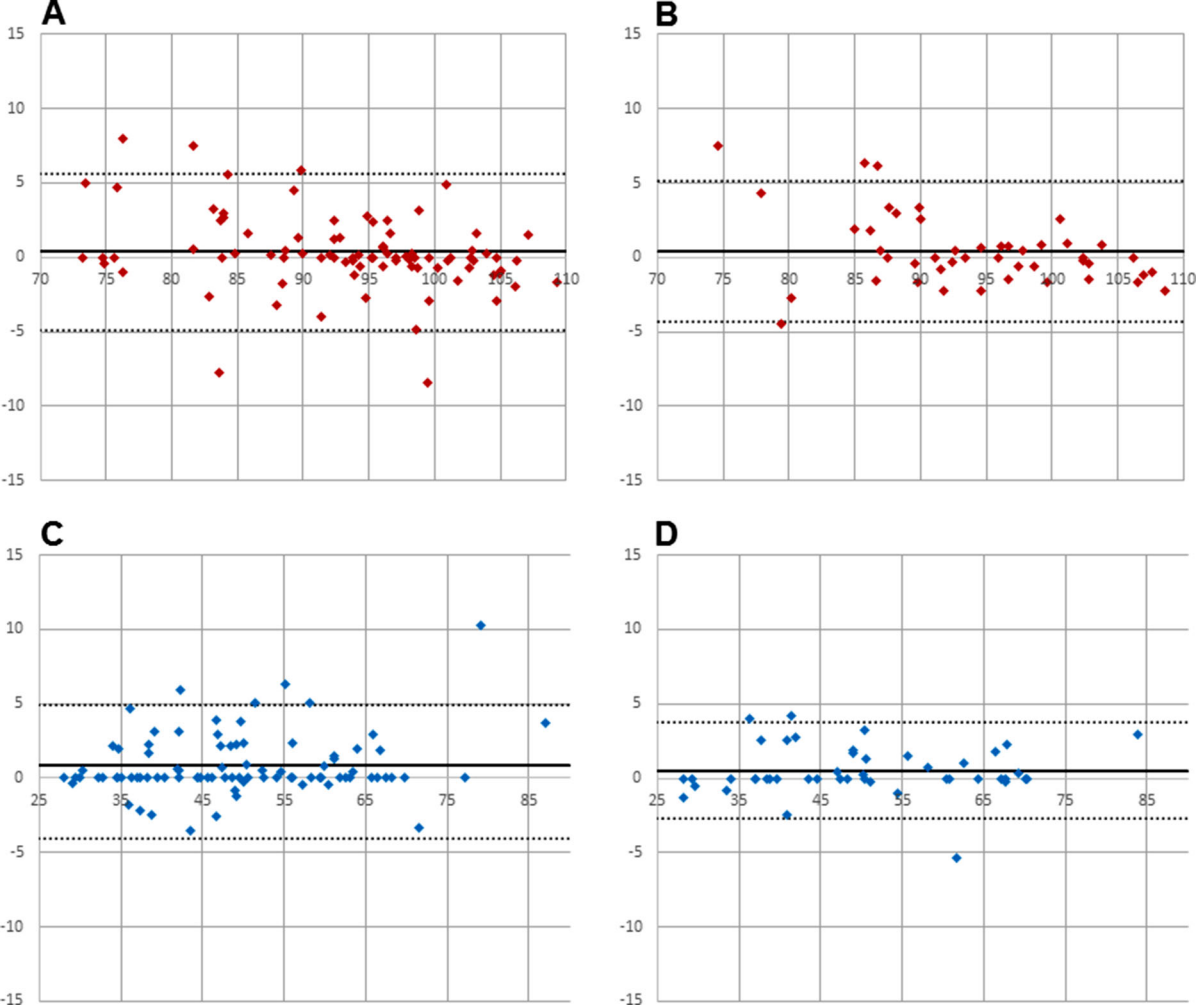
(A) Arteries (30° image: average of all vessels cursing through the measurement annulus vs. average of the four largest vessels; n = 87); (B) arteries (50° image: average of all vessels cursing through the measurement annulus vs. average of the four largest vessels; n = 47); (C) veins (30° image: average of all vessels cursing through the measurement annulus vs. average of the four largest vessels; n = 87); (D) veins (50° image: average of all vessels cursing through the measurement annulus vs. average of the four largest vessels; n = 47).

**Figure 5.**
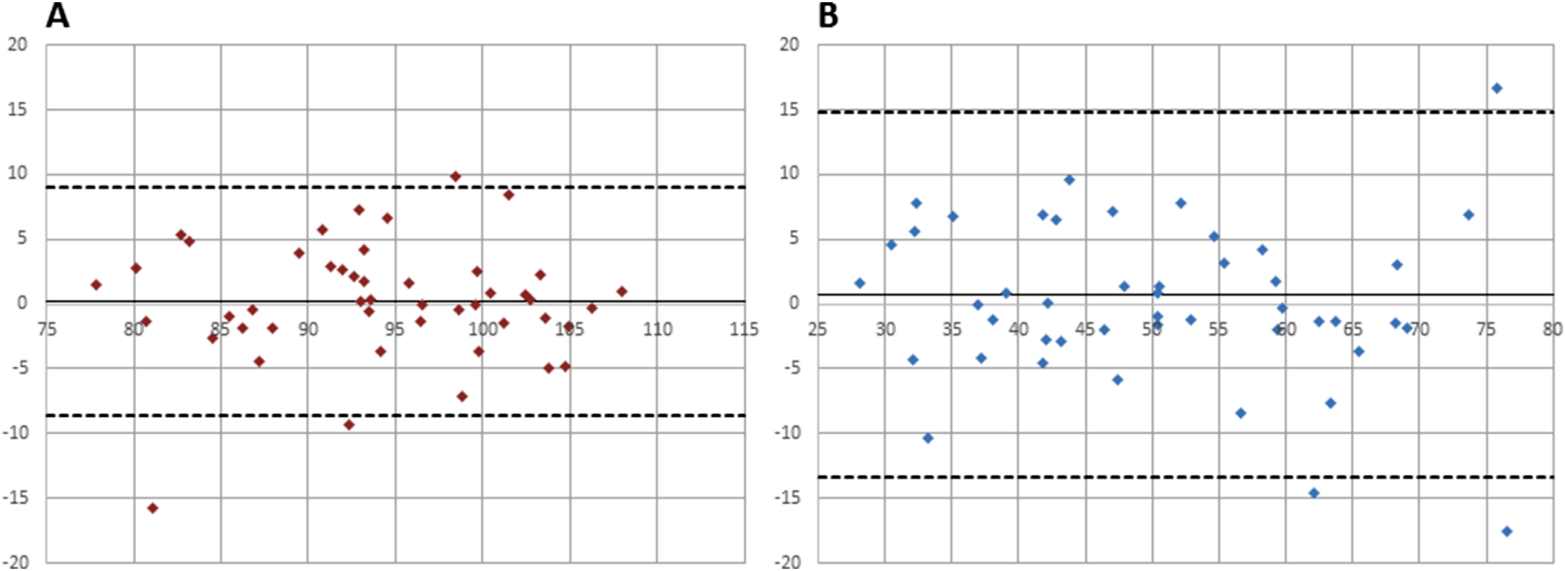
(A) All arteries (30° vs. 50° image; averaged); (B) All veins (30° vs. 50° image; averaged).

**Table 1.** Results of the Bland-Altman-Analyses

Arteries	Bias	Upper Limit	Lower Limit
Protocol a (30° camera angle)	−0.21	5.71	−6.12
Protocol b (30° camera angle)	−0.22	3.82	−4.25
Protocol c (30° camera angle)	0.39	5.64	−4.86
			
Protocol a (50° camera angle)	−0.29	8.34	−8.92
Protocol b (50° camera angle)	−0.33	5.48	−6.14
Protocol c (50° camera angle)	0.43	5.16	−4.30
Protocol d	0.21	9.04	−8.62
**Veins**			
Protocol a (30° camera angle)	−0.31	8.52	−9.14
Protocol b (30° camera angle)	−0.23	5.50	−5.96
Protocol c (30° camera angle)	0.84	4.94	−4.10
Protocol a (50° camera angle)	−0.50	9.24	−10.24
Protocol b (50° camera angle)	−0.51	5.13	−6.15
Protocol c (50° camera angle)	0.51	3.72	−2.70
Protocol d	0.71	14.82	−13.40

**Table 2.** Means and Standard Deviations for each Protocol Assessed

Camera Angle [°]		Mean (+/−SD)	*P* Value
**Arteries**			
30	Protocol a	94.17 (10.48)	0.355
30		94.38 (10.63)	
50	Protocol b	93.99 (10.63)	0.374
50		94.28 (10.99)	
30	Protocol c	93.16 (8.94)	0.182
30		92.77 (9.86)	
50	Protocol c	94.21 (7.71)	0.233
50		93.78 (8.53)	
**Veins**			
30	Protocol a	52.32 (17.85)	0.355
30		52.63 (17.76)	
50	Protocol b	52.39 (17.86)	0.186
50		52.89 (18.42)	
			
30	Protocol c	49.93 (12.81)	**<0.001**
30		49.08 (12.43)	
50	Protocol c	50.29 (13.49)	**0.040**
50		49.78 (13.46)	

## Results

All participants were normotensive and had IOPs within a normal range (9–16 mm Hg). Mean bias was in all instances smaller than 1% oxygen saturation (Table 1). Limits of agreement were smallest when using a measurement protocol, which incorporated calculations of average saturation values rather than using single vessel measurements (Table 1 and Figs. 2^3^,^4^–5).

The results of the paired *t*-tests comparing protocols are illustrated in Table 2. Regardless of camera angle or calculation approach, all arterial vessel oxygen saturation values were comparable. However, the paired *t*-test results for venous oxygen saturation values were significantly different for the 30° and 50° analyses when comparing the approach of including all versus only the four major vessels within the measurement annulus.

## Discussion

Methods and protocols used to clinically assess patients often vary, especially when new devices/methods first become available. Although this makes it difficult to compare studies, it is a necessary process toward the establishment of a standard examination and analysis protocol, or at the very least a set of rules by which different methods/protocols can be applied to arrive at the same clinical conclusion. In this study, we did not assess the agreement between different devices but did assess the measurement and calculation protocols used in the wider research community, which has the strength in that it minimizes technology-based variation. Although our results show a particularly small bias (<1% oxygen saturation) for both arteries and veins, they also highlight the fact that when only using a single vessel measurement the data shows a wider spread of retinal vessel oxygen saturation values. This increase in spread is in part attributable because of the differences found in vessel saturations depending on their location around the optic disc. For a location-specific analysis there might still be interest in using only a single vessel, but considering that there is also variability across individual images it is prudent to employ at least an averaging method across several images of the single vessel under observation.

Although we conducted a paired *t*-tests for all, our protocol comparisons should be emphasized that this does not provide the same insight into the data as the Bland-Altman analysis offers. Two of our paired *t*-tests, namely those comparing protocols employing a vessel averaging approach across the measurement annulus and three images (Table 2), exhibited statistical significance, despite a <1% saturation difference. This significance can be in part attributed to the low average difference between the means and their similar standard deviations. Despite the statistical significance, the observed 1% saturation difference is well below the test-retest reliability of the instrumentation, and there are no established cutoff values or reference data as to what is a clinically meaningful change in saturation, therefore this finding has no clinical relevance.

Bland-Altman plots are widely used to assess agreement between methods and protocols, but the analysis in itself does not state if the agreement is sufficient to use or replace one method/protocol with the other. The main purpose of this type of analysis is to quantify the bias (differences between methods/protocols) and define the range of agreement using 95% confidence intervals of the mean bias.^19^^,^^20^ If the bias is close to zero, then the variability of the differences between the two methods/protocols is mainly derived from the analytical inaccuracy of the methods. However, if the bias is significantly larger (or smaller) than zero this means that on average the second method is producing readings, which are on average the amount of the mean bias larger (or smaller) than those obtained by method one. Thus with respect to the interpretation of the Bland-Altman plot, it is reasonable to say that a bias close to zero is desirable, but to determine if agreement is sufficient with respect to clinical decision-making it is important to establish what agreement interval is sufficiently narrow to do so. The latter can be achieved by considering short-term agreement, day-to-day agreement, interobserver agreement, and other sources that could introduce variability but still allow for the same clinical decision to be made.

As no two individuals have an identical retinal vascular anatomy in regard to size, location and branching pattern of arteries, and veins this means that when using a defined analysis area there can be differences in regard to the number of vessels included for calculation of oxygen saturation measurement. Hence quadrantic differences as described by Palsson et al.^13^ could have an effect on the overall mean depending on how many vessels in total are included. Despite this, when we compared the mean arterial and venous oxygen saturations as calculated by considering all or only the four largest vessels crossing the measurement annulus we found good agreement between both calculations, and our results of mean arterial and venous oxygen saturation parameters show good agreement with previously published data of healthy individuals.^5^^,^^13^^,^^15^^,^^16^^,^^21^^–^^23^ Our aim was to identify if there was a particular analysis protocol, which would yield the smallest bias and spread. These findings support previous work in which single vessel comparisons tend to be more variable compared with average vessel results. In addition, we were able to show that when considering all vessels versus only the four largest vessels, there was little impact in agreement or data spread irrespective of the camera angle used. However, for clinical purposes, it is paramount to use same size images for follow-up measures, as this had the largest impact on overall agreement shown by increase in limits of agreements by a factor of approximately two. This present study only examined retinal vessel oximetry measurements of the major arterial and venous arcades with a minimum size of 70 µ, therefore the results are not applicable to other retinal locations or smaller vessels. Whether averaging across several vessels and between images also yields smaller limits of agreement in other retinal locations other than the ONH remains to be shown. The retinal vessel saturation measurements around the ONH are thought to reflect the amount of incoming and returning oxygen levels and are often used to calculate arterial minus venous (A-V) saturation as a proxy for the amount of oxygen consumed. Increases in oxygen saturation of retinal veins or a decrease in A-V saturation has been largely linked to tissue dysfunction and loss. As loss and/or tissue dysfunction is not always homogenous across the entire retina, it seems plausible to assess vessels linked with specific areas (i.e., quadrants or location specific, such as macula and ONH) separately. Although our results showed the largest limits of agreement for single vessel comparison of two images, this does not necessarily mean that single vessels per se have a larger variance than measurements, which have used multiple images and/or vessels. Averaging across several images, whether this is one single vessel across multiple images or using multiple vessels of a given measurement zone, and averaging these across images to obtain a more stable measurement reflecting incoming/outgoing saturation levels is yielding a lower variance, said variance could also hold crucial information about the metabolic state of the retina. This is because an increased variance could, for example, mean a less stable oxygen delivery leading to intermittent hypoxia, which in turn could lead to slow tissue compromise. Whether the current method has sufficient temporal resolution to capture such mechanisms needs to be established first. We also note that this study is limited compared with most published work using retinal oximetry because our data are from a multiethnic cohort, and hence individuals showed more variability across saturation values, which can be explained in part by their differences in fundus pigmentation. Having said this, our saturation values obtained were all corrected for fundus pigmentation as detailed elsewhere.^5^ However, as the algorithm implemented in the device had been developed using images of white individuals, albeit with different iris colors, there could have still been a residual error, which may explain our slightly larger spread of oxygen saturation values. Although this may explain the larger spread of oxygen saturation values, this would not have impacted on our analyses of agreement as they were carried out using a pairwise approach.

## Conclusions

These results emphasize that including multiple vessels and images to calculate arterial and venous retinal vessel oxygen saturation reduces overall variance. Concordance was worst for single vessel comparisons. For vessels measured in a circular measurement zone around the ONH and have at least 70 µ in diameter, all comparisons (single and multiple vessel calculations) showed a particularly low bias of <1% oxygen saturation. For clinical studies aiming to assess oxygen delivery and return to calculate A-V as a proxy for oxygen consumption, we would recommend to use an analyses approach that includes either all or just the four major vessel arcades passing through the measurement annulus around the ONH, as the comparison between both showed good agreement and concordance. For follow-up studies, we would recommend that only images with the same image size (i.e., camera angle) are used because whereas the bias between saturations obtained with different camera angles was as low as when using the same camera angle, concordance was poor, which in turn means that small changes are likely to be undetectable.

## References

[1] Linsenmeier RA, Zhang HF Retinal oxygen: from animals to humans. *Prog Retin Eye Res*. 2017; 58: 115–151. 28109737 10.1016/j.preteyeres.2017.01.003 PMC5441959

[2] Hoppe-Seyler F Uever die chemischen und optischen eigenschaften des blutfarbstoffs. *Virchows Arch*. 1864; 23: 446–449.

[3] Beach J Pathway to retinal oximetry. *Transl Vis Sci Technol*. 2014; 3: 2. 10.1167/tvst.3.5.2 PMC4164112 25237591

[4] Olafsdottir OB, Eliasdottir TS, Kristjansdottir JV, Hardarson SH, Stefánsson E Retinal vessel oxygen saturation during 100% oxygen breathing in healthy individuals. *PLoS One*. 2015; 10: e0128780. 26042732 10.1371/journal.pone.0128780 PMC4456093

[5] Hammer M, Vilser W, Riemer T, Schweitzer D Retinal vessel oximetry-calibration, compensation for vessel diameter and fundus pigmentation, and reproducibility. *J Biomed Opt*. 2008; 13: 054015. 19021395 10.1117/1.2976032

[6] Stefánsson E, Olafsdottir OB, Eliasdottir TS, et al. Retinal oximetry: metabolic imaging for diseases of the retina and brain. *Prog Retin Eye Res*. 2019; 70: 1–22. 30999027 10.1016/j.preteyeres.2019.04.001

[7] Lim LS, Lim XH, Tan L Retinal vascular oxygen saturation and its variation with refractive error and axial length. *Transl Vis Sci Technol*. 2019; 8: 22. 10.1167/tvst.8.4.22 PMC6685695 31403000

[8] Zheng Q, Zong Y, Li L, et al. Retinal vessel oxygen saturation and vessel diameter in high myopia. *Ophthalmic Physiol Opt*. 2015; 35: 562–569. 26303449 10.1111/opo.12223

[9] Olafsdottir OB, Hardarson SH, Gottfredsdottir MS, Harris A, Stefánsson E Retinal oximetry in primary open-angle glaucoma. *Invest Ophthalmol Vis Sci*. 2011; 52: 6409–6413. 21715353 10.1167/iovs.10-6985

[10] Jørgensen CM, Hardarson SH, Bek T The oxygen saturation in retinal vessels from diabetic patients depends on the severity and type of vision-threatening retinopathy. *Acta Ophthalmol*. 2014; 92: 34–39. 24330421 10.1111/aos.12283

[11] Zong Y, Lin L, Yi C, et al. Retinal vessel oxygen saturation and vessel diameter in retinitis pigmentosa at various ages. *Graefes Arch Clin Exp Ophthalmol*. 2016; 254: 243–252. 25952041 10.1007/s00417-015-3039-6

[12] Todorova MG, Türksever C, Schorderet DF, Valmaggia C Retinal vessel oxygen saturation in patients suffering from inherited diseases of the retina. *Klin Monbl Augenheilkd*. 2014; 231: 447–452. 24771189 10.1055/s-0034-1368236

[13] Palsson O, Geirsdottir A, Hardarson SH, Olafsdottir OB, Kristjansdottir JV, Stefánsson E Retinal oximetry images must be standardized: a methodological analysis. *Invest Ophthalmol Vis Sci*. 2012; 53: 1729–1733. 22395877 10.1167/iovs.11-8621

[14] Sín M, Sínová I, Chrapek O, et al. The effect of pars plan vitrectomy on oxygen saturation in retinal vessels–a pilot study. *Acta Ophthalmol*. 2014; 92: 328–331. 23848230 10.1111/aos.12238

[15] Lasta M, Palkovits S, Boltz A, et al. Reproducibility of retinal vessel oxygen saturation measurements in healthy young subjects. *Acta Ophthalmol*. 2012; 90: e616–e620. 22938808 10.1111/j.1755-3768.2012.02513.x

[16] Told R, Boltz A, Schmetterer L, et al. Method comparison of two non-invasive dual-wavelength spectrophotometric retinal oximeters in healthy young subjects during normoxia. *Acta Ophthalmol*. 2018; 96: e614–e618. 29488329 10.1111/aos.13719

[17] Patel SR, Hudson C, Flanagan JG, Heitmar R The effect of simulated cataract light scatter on retinal vessel oximetry. *Exp Eye Res*. 2013; 116: 185–189. 24056207 10.1016/j.exer.2013.09.004

[18] Heitmar R, Attardo A. The influence of simulated cataract on retinal vessel oximetry measurements. *Acta Ophthalmol*. 2016; 94: 48–55. 26293648 10.1111/aos.12826 PMC6680341

[19] Bland JM, Altman DG. A note on the use of the intraclass correlation coefficient in the evaluation of agreement between two methods of measurement. *Comput Biol Med*. 1990; 20: 337–340. 2257734 10.1016/0010-4825(90)90013-f

[20] Giavarina D Understanding Bland-Altman analysis. *Biochem Med (Zagreb)*. 2015; 25: 141–151. 26110027 10.11613/BM.2015.015 PMC4470095

[21] Schweitzer D, Hammer M, Kraft J, Thamm E, Konigsdorffer E, Strobel J In vivo measurement of the oxygen saturation of retinal vessels in healthy volunteers. *IEEE Trans Biomed Eng*. 1999; 46: 1454–1465. 10612903 10.1109/10.804573

[22] Geirsdottir A, Palsson O, Hardarson SH, Olafsdottir OB, Kristjansdottir JV, Stefansson E Retinal vessel oxygen saturation in healthy individuals. *Invest Ophthalmol Vis Sci*. 2012; 53: 5433–5442. 22786895 10.1167/iovs.12-9912

[23] Palkovits S, Lasta M, Told R, et al. Retinal oxygen metabolism during normoxia and hyperoxia in healthy subjects. *Invest Ophthalmol Vis Sci*. 2014; 55: 4707–4713. 25015353 10.1167/iovs.14-14593

